# Editorial: Novel applications of Bayesian and other models in translational neuroscience

**DOI:** 10.3389/fnins.2024.1373633

**Published:** 2024-04-29

**Authors:** Jacob Raber, Benjamin R. Pittman-Polletta, Reza Rastmanesh

**Affiliations:** ^1^Department of Behavioral Neuroscience, Neurology, and Radiation Medicine, Division of Neuroscience, Oregon National Primate Research Center, Oregon Health and Science University, Portland, OR, United States; ^2^Department of Mathematics and Statistics, Boston University, Boston, MA, United States; ^3^American Physical Society, College Park, MD, United States

**Keywords:** translational neuroscience, Bayesian and other models, data analysis, probabilistic, Bayesian analytics

The task of both the brain and the neuroscientist is to reason about large numbers of variables that are both mutually interdependent and uncertain (i.e., probabilistic). This partly explains why statistical models - and Bayesian models in particular – have been increasingly prominent in both theoretical accounts of brain function and methodologies for analyzing neural data. Bayes' theorem specifies the optimal way to combine prior beliefs with data in probabilistic inference,^1^ offering a powerful tool for reasoning under uncertainty (van Amersfoort et al., 2020). Within the framework of *Bayesian networks*, the values (or rather probability distributions) of multiple variables interrelated through a network of conditional dependencies can be calculated from observational data by successive applications of Bayes' theorem. Bayesian networks can be used as statistical models for a large and general class of dynamical phenomena, and can be constructed using expert knowledge or learned from data through the process of *structure learning*. Recent theories of brain function suggest that perception, cognition, and action can all be fruitfully understood as forms of Bayesian inference, in which an internal generative model of the world is inverted to fit sensory data. This internal generative model can be formalized as a Bayesian network that is dynamic and *hierarchically deep* – i.e., composed of multiple levels of (increasingly abstract) explanatory variables evolving in time. Inversion of this network is believed to be implemented via *predictive processing*, in which brain activity principally encodes the difference between model-generated predictions and sensory data, i.e., *prediction errors*. In perception, the model is changed to match the sensory data, while in action, the sensory data is changed to match the model through so-called *active inference*. In perhaps the farthest-reaching formulation of these hypotheses, the free-energy principle, the brain accomplishes Bayesian inference by performing a gradient descent on free energy. This ensures that the accuracy of the internal model (and its predictions) increases, while its complexity decreases (Bruineberg et al., 2016).

However, while Bayesian, predictive, and statistical models have been proposed as qualitative and quantitative models and tools for basic research, the applications of these models to translational neuroscience have been understudied and underreported. Exceptions include variational Bayesian mixed-effects inference, which has been successfully tested for use in classification studies (Brodersen et al., 2013), and a recently-published multi-task Bayesian compressive sensing approach to simultaneously estimate the full posterior of the CSA-ODF and diffusion-weighted volumes from multi-shell HARDI acquisitions. This Research Topic collects further research applying Bayesian and statistical tools, techniques, and theories to the prediction or anticipation of brain function in humans and animal models under physiological and pathological conditions.

Many of the studies in this Topic employ Bayesian networks (BNs) to analyze and make predictions about neurophysiological data. In Fan et al., structure learning is applied to create a predictive model for ischemic stroke (IS) by discovering a BN linking risk factors to IS in patients with dilated cardiomyopathy (DCM). As Fan et al. point out, a major advantage of BNs is their utility in classifying imbalanced datasets, a common challenge in real-world data. In Carvalho do Nascimento et al., techniques from structure learning for BNs are applied to the discovery of functional connectivity networks in the domain of interpersonal neural synchronization (INS). The proposed two-step network estimation method allows inference of the time-varying probabilistic dependencies between brain regions both within and between subjects. Carvalho do Nascimento et al. demonstrate the utility of their method in the analysis of fNIRS hyperscanning data recorded simultaneously from violinists playing a duet, confirming that one player was leading the other. In Chen, techniques from structure learning are applied to create a data fusion method, called Bayesian Multisource Data Integration, to model the interactions among data sources (i.e., imaging modalities) and behavioral variables. The proposed method constructs a Bayesian network model associating features in each data source with behavioral outcome variables. The generated Bayesian network is transparent and easy to understand. It can be used to understand how behavioral changes depend on features in each data source, and to identify which features synergistically contribute to behavioral outcomes, which are redundant, and which are uninformative.

Thome et al. take the use of Bayesian statistical models for data analysis a step further. They propose a novel use for interpretable latent variable models. These models probabilistically link behavioral observations to an underlying latent process, and have increasingly been used to draw inferences about cognition from observed behavior. The latent process usually connects experimental variables to cognitive computation. While such models provide important insights into the latent processes generating behavior, one important aspect has often been overlooked. They may also be used to generate precise and falsifiable behavioral predictions as a function of the modeled experimental variables. In doing so, they pinpoint how experimental conditions must be designed to elicit desired behavior and generate adaptive experiments. These ideas are exemplified on the process of delay discounting (DD). After inferring DD models from behavior on a typical DD task, the models are leveraged to generate a second adaptive DD task, which elicits 9 graded behavioral discounting probabilities across participants. Models are then validated and contrasted to competing models in the field by assessing the out-of-sample prediction error. They also report evidence for inter-individual differences with respect to the most suitable models underlying behavior. Finally, they outline how to adapt the proposed method to the investigation of other cognitive processes including reinforcement learning.

Priorelli and Stoianov further the application of Bayesian network models of the brain, presenting a normative computational theory of how the brain may support visually-guided goal-directed actions in dynamically changing environments. This theory extends active inference, a theory of cortical processing according to which the brain maintains beliefs over the environmental state, and motor control signals try to fulfill the corresponding sensory predictions. The authors propose that the neural circuitry in the Posterior Parietal Cortex (PPC) compute flexible intentions (Duarte-Carvajalino et al., 2014)—or motor plans from a belief over targets—to dynamically generate goal-directed actions, and develop a computational formalization of this process. A proof-of-concept agent embodying visual and proprioceptive sensors and an actuated upper limb was tested on target-reaching tasks. The agent behaved correctly under various conditions, including static and dynamic targets, different sensory feedbacks, sensory precisions, intention gains, and movement policies; limit conditions were individuated, too. Active inference driven by dynamic and flexible intentions can thus support goal-directed behavior in constantly changing environments, and the PPC might putatively host its core intention mechanism. More broadly, the study provides a normative computational basis for research on goal-directed behavior in end-to-end settings and further advances mechanistic theories of active biological systems.

Mezzetti et al. apply Bayesian models to the analysis of psychometric data, extending their use of generalized linear mixed models (GLMM) and two-level methods in a Bayesian framework. This allows them to apply *a priori* knowledge from the literature and from previous experiments to estimation of psychometric functions, reducing the uncertainty of the parameters through the combination of prior knowledge and the experimental data. Evaluating uncertainties between and within participants through posterior distributions, Mezzetti et al. use a special type of Bayesian model, the power prior distribution, to modulate the weight of the prior, constructed from a first set of data, and use it to fit a second one. Their models estimated the probability distributions of the parameters of interest conveying information about the effects of the experimental variables and their uncertainty, as well as the reliability of individual participants.

The work collected in this Topic also includes translational applications of more general statistical models and approaches. Floyrac et al. used auditory evoked potentials recorded non-invasively during an oddball paradigm in a cohort of 29 post-cardiac arrest anoxic comatose patients to predict return to consciousness and good neurological outcomes. By extracting features from the standard and the deviant auditory stimulations independently and using machine learning to cluster patients within the two-dimensional space determined by these features, they were able to predict patients' neurological outcomes with a sensitivity of 0.83 and an accuracy of 0.90, even when using data only from one electrode. Ren et al. constructed a diagnostic model for cognitive impairment, a common disorder in patients with epilepsy, using the clinical and the phase locking value functional connectivity features of the electroencephalogram (EEG). Yoshiiwa et al., motivated by electroencephalographic studies of working memory demonstrating cortical activity and oscillatory representations without clarifying how the stored information is retained in the brain, measured scalp electroencephalography data while participants performed a modified n-back working memory task. They then calculated the current intensities from the estimated cortical currents by introducing a statistical map generated using Neurosynth as prior information. Their results indicate that the representation of executive control over memory retention may be mediated through both persistent neural activity and oscillatory representations in the beta and gamma bands over multiple cortical regions that contribute to visual working memory functions. Yazawa et al. created an arterially perfused *in situ* brainstem and spinal cord preparation that allowed them to investigate functional interactions in the CNS from the neonatal to adult period, bypassing the technical limitations on the spatial and temporal scope of *in vitro* neonatal rodent spinal cord preparations imposed by low oxygen tension in deep tissues. Using their novel preparation, they explored whether the absence of interferon regulatory factor 8 (IRF8) – which affects behavior and modulates Alzheimer's disease progression in a mouse model – influences the development of lumbar central pattern generator (CPG) networks in mice of all ages. Finally, Mount et al. explored how autism spectrum disorder (ASD) risk genes influence neural circuit computation during behavior by performing large-scale cellular calcium imaging from hundreds of individual CA1 neurons simultaneously in transgenic mice with total knockout of the X-linked ASD-risk gene *NEXMIF* (neurite extension and migration factor). As *NEXMIF* knockout in mice led to profound learning and memory deficits, they examined the CA1 network during voluntary locomotion, a fundamental component of spatial memory. They found that in wild-type mice the CA1 network desynchronizes during locomotion, consistent with increased network information coding during active behavior. Upon *NEXMIF* knockout, the CA1 network is over-synchronized regardless of behavioral state and fails to desynchronize during locomotion, highlighting how perturbations in ASD-implicated genes create abnormal network synchronization that could contribute to ASD-related behaviors.

In conclusion, it is our hope that the work collected in this Topic will serve as a basis for future studies exploring the potential application of Bayesian and other models in Translational Neuroscience.

## Author contributions

JR: Conceptualization, Writing—original draft, Writing—review & editing. BP-P: Conceptualization, Writing—original draft, Writing—review & editing. RR: Conceptualization, Writing—original draft, Writing—review & editing.

## Footnotes

^1^Bayes' theorem states that the *conditional probability* of some occurrence *A* given observed data *B, P*(*A*||*B*), is proportional to the product of the *prior probability* of the event, *P*(*A*), and the *likelihood* of the observation given the event, *P*(*B*||*A*). We can think of the conditional distribution *P*(*B*||*A*) as a *generative model* of the data, which we *invert* to calculate the *posterior probability P*(*A*||*B*).
